# The Genus *Artemisia*: A 2012–2017 Literature Review on Chemical Composition, Antimicrobial, Insecticidal and Antioxidant Activities of Essential Oils

**DOI:** 10.3390/medicines4030068

**Published:** 2017-09-12

**Authors:** Abhay K. Pandey, Pooja Singh

**Affiliations:** Bacteriology & Natural Pesticide Laboratory, Department of Botany, DDU Gorakhpur University Gorakhpur, Uttar Pradesh 273009, India; abhaykumarpandey.ku@gmail.com

**Keywords:** *Artemisia*, essential oil, chemical composition, antimicrobial, insecticidal, antioxidant

## Abstract

Essential oils of aromatic and medicinal plants generally have a diverse range of activities because they possess several active constituents that work through several modes of action. The genus *Artemisia* includes the largest genus of family Asteraceae has several medicinal uses in human and plant diseases aliments. Extensive investigations on essential oil composition, antimicrobial, insecticidal and antioxidant studies have been conducted for various species of this genus. In this review, we have compiled data of recent literature (2012–2017) on essential oil composition, antimicrobial, insecticidal and antioxidant activities of different species of the genus *Artemisia*. Regarding the antimicrobial and insecticidal properties we have only described here efficacy of essential oils against plant pathogens and insect pests. The literature revealed that 1, 8-cineole, beta-pinene, thujone, artemisia ketone, camphor, caryophyllene, camphene and germacrene D are the major components in most of the essential oils of this plant species. Oils from different species of genus *Artemisia* exhibited strong antimicrobial activity against plant pathogens and insecticidal activity against insect pests. However, only few species have been explored for antioxidant activity.

## 1. Introduction

Aromatic and medicinal plants are important sources of secondary metabolites, which have a wide range of applications in control of plant and human diseases, cosmetics, as well as in the pharmaceutical industry [1]. In the plant kingdom, family Asteraceae is endowed with essential oil-yielding plants, and among these plants, the genus *Artemisia* occupies top position for its bio-prospection. The genus consists of small herbs and shrubs, found in northern temperate regions and comprises of about 500 species from South Asia, North America and European countries [2]. Species of the genus are called by the common names mugwort, wormwood and sagebrush. Due to presence of terpenoids and sesquiterpene lactones, most of the species possess strong aromas and bitter tastes, which discourage herbivory, and may have had a selective advantage [3]. These species have wide and varied applications in plant and human disease control and in the pharmaceutical industry. There are several species of *Artemisia* that have been investigated as antimicrobial, antioxidant, cytotoxic, insecticidal, repellent and anticonvulsant agents [4,5]. Although a review on the genus *Artemisia* was published by Abad et al. [2] on the chemical composition, ethanopharmacological and biocidal activity of essential oils, they took the data from 2000–2011, and mainly focused on human pathogens. Recently, another review compiled by Al-Snafi [6], which focused only on *A. campestris*, revealed several pharmacological activities, such as antimicrobial, antioxidant, cytotoxic, insecticidal, antivenomous, and many other pharmacological effects. In this review, we have compiled the data from 2012–2017 on chemical composition, antimicrobial, insecticidal and antioxidant activities of *Artemisia* species. Regarding antimicrobial and insecticidal activities of essential oils, here, we have mainly focused on pathogens and pests of plants and, since Abad et al. [2] did not describe earlier literature on antimicrobial and insecticidal activities of *Artemisia* oils on same aspects and also antioxidant activity, so in this review we have also covered the literature from 2000 onwards on these aspects.

## 2. Chemical Composition of Essential oils of *Artemisia* Species

Plant essential oils are volatile in nature and consist of a complex mixture of monoterpenes and sesquiterpenes, which give strong odor to the essential oils. These essential oils are extracted from plants by various methods such as steam or hydro-distillation methods and are frequently being used in the natural product laboratory [7]. Essential oils are composed of more than 60 different components in different concentrations; among them few have higher amounts of composition. From time to time, the chemical composition of essential oils of the genus *Artemisia* has been studied by researchers from the different regions of the world. The essential oil composition of genus *Artemisia* investigated during 2012–2017 is reported in Table 1. The table shows that investigator used leaf, stem, areal part and inflorescence for essential oil extraction and GC and GC/MS methods for the chemical analysis. 1, 8-Cineole, beta-pinene, thujone, artemisia ketone, camphor, caryophyllene, camphene and germacrene D were the major components reported in the essential oils of *Artemisia* species (Table 1). The table also shows that the composition of essential oil of the same species varied in different investigations depending upon a change of geographical origin. Variation in the volatile components of these plants may occur during plant ontogeny or growth at different altitudes. However, few chemical constituents were restricted to limited species. For instance, methyl chavicol was only reported in higher amounts in *A. dracunculus*, piperitone in *A. judaica,* capillene in *A. stricta* and chamazulene in *A. arborescens* L, artedouglasia oxide in *A. stelleriana*. Most of the investigations into the chemical composition of essential oils were from Iran, followed by India and China.

## 3. Antimicrobial Properties of *Artemisia* Essential Oils

The interest in using essential oils as an antimicrobial agent is increasing mainly due to their natural origin, wide spectrum of activity and their GRAS (Generally Recognized as Safe) status. Since earlier reviews published on the *Artemisia* oils only described bioactivity against human pathogens, here we have illustrated literature from 2000 onwards on *Artemisia* oils only against plant pathogens. Essential oils from the *Artemisia* species have been explored for their antimicrobial properties against several bacterial and fungal plant pathogens by using different methods such as the disc diffusion method, agar dilution method, poison food method and inverted Petri plate method depending upon the nature of fungal and bacterial species [74]. In our laboratory bioassay, *A. nilagirica* oil performed potent results with postharvest pathogens of table grapes. Oil exhibited 100% mycelia inhibition against *Aspergillus flavus*, *A. niger* and *A. ochraceus* and 0.29 and 0.58 μL/mL fungistatic and fungicidal values, respectively, were noticed for all the fungal species. Oil (1.6 μL) completely suppressed the growth and mycotoxin (AFB1 and OTA) secretion of aflatoxigenic and ochratoxigenic strains of *Aspergillus*. Fumigation of oil (300 μL) was found to protect 1 kg of table grapes and enhanced the shelf life for up to 9 days. Thus, this oil can be used as grape protectant from fungal spoilage [75]. Sati et al. [18] examined in the laboratory that *A. nilagirica* oil is effective against root rot pathogens with ED_50_ (effective dose) values against *Rhizoctonia solani*, *Sclerotium rolfsii* and *Macrophomina phaseolina* were 85.75, 87.63 and 93.23 mg/L, respectively. This oil also showed fungicidal activity against *Colletotrichum fragariae*, *C. gloeosporioides*, and *C. acutatum* and *Artemisia caerulescens* subsp. *densiflora* (Viv.) oil against *Aspergillus*, *Alternaria* and *Fusarium* species [26,76], whereas *A. maritima* oil has poor mycelial growth inhibition. In the three tested *Artemisia* (*A. scoparia*, *A. sieberi* and *A. aucheri)* oils against soil-borne pathogens from Iran, *A. aucheri* and *A. sieberi* oils proved strong antifungal inhibitors with 41.406 μL/L EC_50_ (effective concentration) for *A. aucheri* oil against *Rhizoctonia solani*, while *A. sieberi* oil showed 121.798 μL/L EC_50_ with MIC value (minimum inhibitory concentration) 250 μL/L against *R. solani*. *Artemisia sieberi* oil was also fungistatic against *Tiarosporella phaseolina* (1000 μL/L), *Fusarium moniliforme* (750 μL/L) and *F. solani* (750 μL/L) with EC_50_ values 203.419, 211.072 and 188.134 μL/L, respectively [77]. Essential oils of *A. arborescens* also reported as fungicidal agent against *R. solani* at 12.5 μL/20 mL [22]. Essential oil isolated from *A. absinthium* from different geographical regions showed significant antifungal activity (ED_50_ 0.5 μg/mL) against *F. oxysporum* and *F. solani* [78]. All these plant pathogens have a wide range of hosts including chick pea, mungbean, urdbean, soyabean etc., so their study suggests that these oils can be used as seed treatment for the control of these phytopathogenic fungi infecting agricultural crops. In the laboratory bioassay of Badawy and Abdelgaleil [79], *A. monosperma* oil was examined as a strong mycelial growth inhibitor of *Alternaria alternata*, *Botrytis cinerea*, *F. oxysporum* and *F. solani* and EC_50_ values reported were 54, 111, 106 and 148 mg/L, respectively. However, they noticed that oils of *A. judaica* and *A. monosperma* caused highest spore germination inhibition of *F. oxysporum* at EC_50_ values 69 and 62 mg/L, respectively*.* The growth of *Sclerotinia sclerotiorum*, a crown rot pathogen was inhibited by *A. santonica*, *A. pontica*, *A. annua*, *A. austriaca*, *A. dracunculus*, *A. lerchiana*, *A. vulgaris* and *A. vulgaris* var. *pilosa* oils at MIC of 2400 μL/L. However, *A. abrotanum* showed 1200 μL/L MIC value against *S. sclerotiorum*, while *A. scoparia* did not exhibit complete mycelial inhibition [80]. Some *Artemisia* oils showed weak antifungal activity against plant pathogens i.e., *A. proceriformis* oil showed a poor efficacy (MIC100 > 1.5 mg/mL) on *Septoria glycine* and other phytopathogens tested [57] which shows that this oil cannot be recommended as a plant protection product against said phytopathogens. This strong and poor effect of essential oil of different species of the same genus may be due to chemical composition and their synergistic effect. In another study, *A. campestris* oil showed a potent antifungal agent against *F. graminearum*, *Penicillium citrinum*, *P. viridicatum* and *Aspergillus niger*. The MICs reported were 1.25 μL/mL (*v*/*v*) for *F. graminearum*, while MFC for all fungal species exceeded 20 μL/mL [53]. The MIC of *A. stricta* oil was reported as 0.625 mg/mL against *A. flavus* followed by *A. niger* and *Sporothrix schenckii* [52]. Similarly, essential oils of *A. absinthium* showed 84 and 91 μg/mL MIC values for *P. chrysogenum* and *A. fumigatus* respectively [23].

In addition to essential oil screening, chemical compounds isolated from different species of *Artemisia* have also been evaluated against plant pathogens [81]. 5-phenyl-1,3-pentadiyne and capillarin isolated from *A. dracunculus* oil showed fungicidal activity against *C. fragariae*, *C. gloeosporioides*, and *C. acutatum* [82]. Dadasoglu et al. [74] assessed some chemical constituents like camphor, caryophyllene oxide, linalool, 1, 8-cineole, teminen-4-ol, borneol and α-terpineol isolated from *A. absinthium*, *A. santonicum* and *A. spicigera* oils against plant pathogenic bacteria and fungi. The MIC value of linalool was in the range of 50–110 mg/mL, terpinen-4-ol, 60–110 mg/mL for *Xanthomonas campestris* pv. *vitians* RK-Xcvi; α-terpineol-8, 60–70 mg/mL for *Pseudomonas cichorii* RK-166, *P. huttiensis* RK-260, *P. syringae* pv. *syringae* RK-204 and *X. axonopodis* pv. *vesicatoria*. Other chemical compounds such as caryophyllene oxide, bomeol, camphor and 1, 8-cineole did not show activity against any of the pathogens. Additionally, camphor, 1, 8-cineole and chamazulene isolated from *A. absinthium* oil from the Turkish population has been described as an effective fungicidal agent against wilt fungi *F. solani* and *F. oxysporum* [83] and from Uruguay the same species rich in thujone showed potent fungicidal activity against *Alternaria* sp. and *Botrytis cinerea* [84]. Thus, efficacy of *Artemisia* oils may be due to the presence of these chemical constituents and these chemical constituents can be used as potential antimicrobial agents against said pathogens [74]. Mycologists assumed that these chemical constituents present in the essential oils cause degeneration of fungal hyphae result in potent antifungal activity. Chemical compounds of essential oils dissolved in the membranes and therefore increase the permeability of the cell membrane, resulting in membrane swelling and reduction of membrane function [85]. Additionally, essential oils penetrate the cell walls of fungi due to their lipophilic property therefore affecting the enzymes involved in cell wall synthesis reactions, thus causes morphological changes in fungi which further lead to the lysis of the fungal cell wall [86].

## 4. Efficacy of *Artemisia* Oils against Insect Pests

Research has been conducted to see the effect of *Artemisia* oils against insect pests of agricultural crops, especially pests of stored products, in order to search out their efficacy as a repellent, insecticidal agent or antifeedant. From several national and international research institutions, investigators evaluated the essential oils from different species of genus *Artemisia* against storage and field insect pests. *A. arborescens* essential oil exhibited insecticidal effects against stored grain pest *Rhyzopertha dominica* at the dose of 50 μL in Petri dish [22]. A 37 μL/L and 24 h of exposure time of *A. sieberi* oil was sufficient to cause 100% mortality of *Callosobruchus maculatus*, Sitophilus oryzae and *Tribolium castaneum.* LC_50_ (lethal concentration) values estimated for oil were 1.45 μL/L against *C. maculatus*, 3.86 μL/L against *S. oryzae* and 16.76 μL/L against *T. castaneum* [87]. In a filter-paper arena test, *A. vulgaris* oil had a very strong repellent activity against *T. castaneum* adults at a 0.6 μL/mL (*v*/*v*). In fumigation tests, 8.0 μL/mL dose of *A. vulgaris* oil exhibited 100% mortality of *T. castaneum* adults; mortality of larvae achieved was only 53%. A 20 μL/L air and a 96 h exposure of the oil showed 100% ovicidal activity; however, at a higher dose (60 μL/L) of this oil no larvae, pupae and adults were observed [88]. In fumigant toxicity test, 11.2 and 15.0 mg/L air LC_50_ values were reported against *Sitophilus zeamais* adults, while in a contact toxicity test LD_50_ (lethal dose) were 55.2 and 112.7 mg/adult for *A. lavandulaefolia* and *A. sieversiana* oils, respectively [89]. In another study [90], they found LC_50_ 5.31 and 7.35 mg/L, respectively for *A. capillaris* and *A. mongolica* essential oils against *S. zeamais* adults in fumigant bioassay, while in contact bioassay LD_50_ values were 105.95 and 87.92 μg/adult, respectively. Again, *A. scoparia* essential oil achieved 100% mortality of *C. maculatus* at 37 μL/L air (24 h) in fumigant bioassay with LC_50_ for the oil was 1.46 μL/L against *C. maculatus* and 2.05 μL/L air against *S. oryzae* and *T. castaneum* [91]. Similarly, 80–90% mortality of granary weevil, *S. granarius* (L.) was reported due to *A. absinthium*, *A. santonicum* and *A. spicigera* oils at a dose of 9 μL/L air after 48 h of exposure [92]. Against *S. oryzae*, *A. princeps* oil when mixed with *Cinnamomum camphora*, it showed strong repellent effect in 1:1 ratio and 1000 μg/mL of dose exhibited insecticidal action [93]. LC_50_ value for *A. vestita* oil against *S. zeamais* in fumigant bioassay was 13.42 mg/L air, while LD_50_ reported was 50.62 mg/adult in contact bioassay [94]. Later on, using same insect, they [8] determined 6.29 and 17.01 mg/L air LC_50_ of *A. giraldii* and *A. subdigitata* oils in fumigant test and that of corresponding LD_50_ 40.51 and 76.34 μg/adult, in a contact test. EC_50_ for *A. annua* oil was estimated to be 2.6 and 4.1 μL/mL against *C. maculatus* and *T. castaneum*, respectively [95], and LD_50_ value of *A. rupestris* oil was 414.48 μg/cm^2^ against *Liposcelis bostrychophila* and *L. bostrychophila* and 6.67 mg/L air LC_50_ against *L. bostrychophila* [96]. This oil has also been proved as an effective insecticide against larval, pupal and adult stages of *Helicoverpa armigera* [97]. *Plodia interpuntella*, a polyphagous insect pest of different stored products worldwide, is found to be controlled by *A. khorassanica* essential oil (LC_50_: 9.6 μL/L air) with lethal time reported at 2.07 h [98]. Sharifian et al. [99] found that *C. maculatus* was more susceptible (LC_50_ 52.47 μL/L air) and *T. castaneum* was more tolerant (LC_50_ 279.86 μL/L air) towards *A. vulgaris* essential oil after 24 h of exposure. Respective LD_50_ and LC_50_ values of *A. argyi* essential oil determined by Zhang et al. [38] were 6.42 μg/adult and 8.04 mg/L air against *Lasioderma serricorne* adults. Their other report on *A. stolonifera* oil [45] showed LD_50_ 8.60 μg/adult against *T. castaneum* and 12.68 μg/adult against *L. serricorne*. The oil showed 1.86 mg/L air LC_50_ value in fumigant test against *T. castaneum*. Liu et al. [36] reported that *A. frigida* essential oil exhibited 17.97 μg/adult and 254.38 μg/cm^2^ LD_50_ in contact toxicity test and 69.46 and 1.25 mg/L air LC_50_ in fumigant test against adults of *S. zeamais* and *L. bostrychophila*, respectively. In contact toxicity, the corresponding LD_50_ values of *A. absinthium* and *A. herba*-*alba* oils against *T. castaneum*, red flour beetle reported were 0.209 and 7.432 μL/L air [100]. In their further study with *Oryzaephilus surinamensis* LC_50_ and LD_50_ values of *A. herba*-*alba* and *A. absinthium* reported in fumigant and contact toxicity bioassay were 30.22 and 0.209 μL/L, respectively [100]. Recently, Liang et al. [101] reported insecticidal activity of *A. anethoides* oil by contact and fumigant tests against *T. castaneum* (LD_50_ 28.80 μg/adult and LC_50_ 13.05 mg/L air, resp.) and *L. serricorne* (LD_50_ 24.03 μg/adult and LC_50_ 8.04 mg/L air, resp.) adults.

Researchers also tested chemical constituents extracted from different species of *Artemisia* in order to make the botanical insecticides with a single and effective constituent. *T*rans**-ethyl cinnamate (LD_50_ 0.37 μg/larva) isolated from *A. judaica* oil was more potent than piperitone (LD_50_ 0.68 μg/larva) against *Spodoptera *littoralis** and also both these compounds caused complete inhibition of feeding activity at 1000 μg/mL [81]. 1,8-cineole and terpinen-4-ol (extracted from *A. absinthium*, *A. santonicum* and *A. spicigera* oils) were more effective against *S. granaries* with 100% mortality at 0.5, 0.75 and 1.0 μL/L air doses after 12 h of exposure [92]. Similarly, among chemical constituents of *A. mongolica* essential oil, 4-terpineol exhibited strongest contact toxicity (LD_50_ 8.62 μg/adult) against *L. serricorne* adults and camphor and alpha-terpineol in fumigant toxicity (LC_50_ 2.91 and 3.27 mg/L air, resp.) [41]. α-Terpinyl acetate (LD_50_ 92.59 μg/cm^2^) of *A. rupestris* oil showed more contact toxicity than α-terpineol (140.30 μg/cm^2^), 4-terpineol (211.35 μg/cm^2^), and linalool (393.16 μg/cm^2^) against book lice *L. bostrychophila* infesting stored cereals [96]. Some chemical constituents of *A. argyi* oil such as camphor (11.30 μg/adult), eucalyptol (15.58 μg/adult), β-caryophyllene (35.52 μg/adult) and β-pinene (65.55 μg/adult) exhibited more toxicity against *L. serricorne* adults having lower LD_50_ values than that of α-terpinyl acetate, 4-terpineol, and linalool isolated from *A. rupestris* oil [37]. In their fumigant toxicity test eucalyptol (LC_50_ 5.18 mg/L air) and camphor (LC_50_ 2.91 mg/L air) had more toxicity than β-pinene (LC_50_ 29.03 mg/L air). Essential oil of *A. ordosica* possessed less toxicity (LC_50_ 18.65 mg/L air) against *T. castaneum* adults than its chemical constituents capillene, capillin, capillinol, *cis*-dehydromatricaria ester (LC_50_ 4.06 to 6.16 mg/L air) tested individually, however, among the essential oil and compounds tested, capillin showed strong repellency (100%) at 62.91, 12.58 and 2.52 μL/cm^2^ after 2 h of exposure [102]. This revealed that the toxic properties of the oil could be attributed to the synergistic effects of its diverse major and minor components. All these results evidence that essential oils from these species of *Artemisia* oils and their constituents can be used in the formulation of botanical insecticides against the said insects for the long-term preservation of food commodities infested by these insects. The mechanism behind the insect mortality in the contact toxicity test is that the volatiles penetrate in the insect body via the respiratory system and result in abnormal breathing, which leads to asphyxiation and finally the death of insects [103]. During fumigant application, main target sites of essential oils and their constituents in insects is the octopaminergic system. When insects are exposed to the essential oils, a breakdown of the nervous system of insects occurs [104] which lead to the blockage of the nerve impulse, later paralysis and then death of the insects occurs.

## 5. Antioxidant Activity

Essential oils and chemical constituents of several *Artemisia* species have been investigated in the laboratory to protect against oxidative damage by inhibiting or quenching free radicals and reactive oxygen species. They have been proved as alternative antioxidants of synthetics. The antioxidant properties of the oils were assessed by several methods such as β-carotene bleaching (BCB) test, the 2,20-diphenyl-1-picrylhydrazyl (DPPH) radical scavenging method, thiobarbituric acid reactive species (TBARS), Trolox equivalent antioxidant capacity assay (TEAC I-III assay), Total radical-trapping antioxidant parameter assay (TRAP assay), N,N-dimethyl-p-phenylendiamine assay (DMPD assay), 2,2′-Azinobis 3-Ethyl-benzothiazoline-6-Sulphonate (ABTS), 2,2-diphenyl-l-picrylhydrazyl assay (DPPH assay), Photochemiluminescence assay (PCL assay) and Ferric reducing ability of plasma assay (FRAP assay) [105]. We assessed the antioxidant activity of *A. nilagirica* essential oil in our laboratory and found that the oil significantly inhibited radical cation formation, with 15.729 μL IC_50_ (Inhibitory concentration) and 13.539 μL IC_50_ preventing the bleaching of β-carotene [106]. While this oil exhibited higher antioxidant activity in the experiment of Sandip et al. [33] in the DPPH (IC_50_ 6.72 μg/mL) test, they reported the *A. chamaemelifolia* essential oil as weak antioxidant. Oil of *A*. *scoparia* induced secondary metabolites production in root cells viz., scavenging enzymes—superoxide dismutase, catalase, ascorbate and guaiacol peroxide and was phytotoxic to root growth causing its inhibition [107]. The *A. annua* essential oil (IC_50_ 27.07 mg/mL) was able to reduce the stable violet DPPH radical to the yellow DPPH-H, reaching 50% of reduction. However, IC_50_ reported in ABTS method was 5.97 mg/mL lower than that of DPPH method. This oil was also 50% able to reduce the ferric ions to ferrous ions (Fe^2+^) at 127.17 mg/mL [11]. This oil showed 18% antioxidant activity of the reference compound (tocopherol) [108]. Phenolic compounds present in the *A. campestris* essential oil contributed its major antioxidant activity, where 47.66 μg/mL EC_50_ was reported in radical scavenging activity, 5.36 μg/mL in FRAP, 0.175 μg/mL in superoxide scavenging activity and 0.034 μg/mL in OH scavenging activity [109]. Thus, this oil can be used as an antioxidant in the pharmaceutical industry.

The pronounced antioxidant activity may be due to the phenolic constituents. *A. campestris* oil showed maximal DPPH activity at dose of 2 mg/mL [110], however, *A. herba*-*alba* oil showed strong DPPH activity (IC_50_ 6 μg/mL) than ABTS assay (IC_50_ 40 μg/mL) [72]. In another study, IC_50_ values of *A. turanica* oil reported were 7.00 mg/mL, 9.69 μg and 14.63 μg, in DPPH, nitric oxide and superoxide anion radicals, respectively. The oil showed ferrous-ion chelating activity at 16.97 μg of IC_50_ [59]. Ali et al. [111] reported that 0.005 mg/mL of ethyl acetate fraction of *A. macrocephala* oil showed 121.5% radicle scavenging activity. However, essential oil of *A. deserti* exhibited more antioxidant activity by DPPH free radical scavenging method (57.2%) than that of β-carotene bleaching test (50%) [17]. In the β-carotene method, *A. dracunculus* oil also showed 50% scavenging activity [21]. On the contrary, essential oils from *A. absinthium*, *A. biennis*, *A. cana*, *A. dracunculus*, *A. frigida*, *A. longifolia* and *A. ludoviciana* from Western Canada showed poor antioxidant activity in both the β-carotene/linoleate model and DPPH radical scavenging tests [112]. In addition, the antioxidant and DPPH radical scavenging activities of camphor and 1, 8-cineole isolated from *Artemisia* species were determined in vitro [113]. Singh et al. [114] reported more IC_50_ (146.3 μg/mL) of *A. scoparia* than that of the antioxidant BHT (140.9 μg/mL) in DPPH bioassay. The residue essential oil also scavenged OH with an IC_50_ of 145.2 μg/mL in the Fenton reaction using a deoxyribose assay. However, unlike scavenging of OH, residue essential oil exhibited a decreased scavenging activity towards H_2_O_2_ (IC_50_ 270.1 μg/mL). They also reported that OH scavenging activities of citronellal and citronellol (25–200 μg/mL) were 8–34 and 11–55%, respectively. For the *A. afra* oil, 50% DPPH radicle scavenging inhibition was reported at 1.1 μL/mL, while it increased for *A. abyssinica* (28.9 μL/mL) oil. In lipid peroxidation bioassay only 0.09 μL/mL of oil is required for 50% inhibition [115]. From Tunisia, Riahi et al. [31] reported the variable IC_50_ values (28.2 and 46.5 g/mL of leaf and flower oils, resp.) in *A. absinthium* oil. Additionally, essential oils from leaves (595.26 mol Fe^2+^/L) and flowers (286.42 mol Fe^2+^/L) also exhibited significant ferric-reducing antioxidant activity. From Serbia, *A. annua* oil showed 50% scavenging of radicle cations at 2.90 μg/mL in DPPH bioassay, and 50% antioxidant activity at 0.640 μg/mL in ABTS assay [24]. However, oil did not show superoxide-scavenging activity. IC_50_ values for the chemical constituents in DPPH and ABTS methods reported were 4.00 and 1.79 μg/mL for Artemisia ketone, 87.0 and 30.1 μg/mL for α-pinene, 47.9 and 6.46 μg/mL for 1,8-Cineole, and 34.4 and 23.6 μg/mL for camphor, respectively. Mohammadi et al. [116] showed that *A. absinthium* essential oils extracted before flowering stage exhibited strong DPPH activity (EC_50_ 3.307 mg/mL) than that of the oils extracted at flowering (EC_50_ 4.11 mg/mL), and after flowering stage (EC_50_ 4.26 mg/mL). This may be due to presence of effective compounds such as sabinene, beta-pinene, alpha-phellandrene, p-cymene, and chamazulene which were more (58.36%) before flowering stage than that of at flowering (48.98%) and after flowering (53.99%). This may be also due to synergistic effect of the compounds [62,117].

## 6. Conclusions

Among herbal plants of the world, genus *Artemisia* biological activity is comparatively less explored against plant pathogens and insect pests. This genus is represented by more than 40 species, especially in the tropics. This review covers the chemical composition of essential oils from different geographical regions where a significant difference in the composition of different species of the same genus is observed. Major components consisted of several terpenes, terpenoids and phenolic compounds; and 1, 8-cineole, beta-pinene, thujone, artemisia ketone, camphor, caryophyllene, camphene and germacrene D were dominant in several species. The different *Artemisia* oils and their compounds have been reported as effective antimicrobial, insecticidal and antioxidant agents. Some oils also exhibited poor to moderate potency against pests and pathogens. Antioxidant activity found in oils is basically due to presence of phenolic compounds. The information summarized here is intended to serve as a reference tool to people in the field of plant protection and natural products chemistry. Although the current review focuses on the antimicrobial role of *Artemisia* essential oils against phytopathogens, it has also shown promising results against several human and animal pathogens. Recently this genus has attracted attention of the world when it commanded a Nobel prize regarding its use in traditional medicine for combating malaria. Although preliminary studies have been done on several species of *Artemisia* regarding its antimicrobial, antioxidant, insecticidal properties, elaborate bioprospection on its probable bioactivites against plant pathogens and pests is needed at field level. Recent times are desperate times where research interest has shifted towards exploration of natural compounds, especially for human welfare. More accurate reporting and data analysis is still needed. Other major issues such as mammalian toxicity, residual toxicity, phytoxicity and legal regulations/obligation and its long-term physiological and ecological effects of the effective oils need to be answered.

## Figures and Tables

**Table 1:** A literature report from 2012–2017 on chemical composition of *Artemisia* oils from different geographical regions (Plant part: AP: aerial parts; F: flowers; FH: flower-heads; L: leaves; B: Buds)

Plant Species	Parts Used	Major Components (%)	Country	Ref.
**Chemical Analysis in 2012**				
*Artemisia giraldii*, *A. subdigitata*	AP	*A. giraldii*: β-Pinene (13.18), iso-elemicin (10.08), germacrene D (5.68), 4-terpineol (5.43), (Z)-β-ocimene (5.06). *A. subdigitata*: 1, 8-Cineole (12.26), α-curcumene (10.77), β-pinene (7.38), borneol (6.23), eugenol (5.87)	China	[8]
*A. monosperma*	L & S	S: β-Pinene (50.3), α-terpinolene (10.0), limonone (5.4), α-pinene (4.6), L: β-Pinene (36.7), α-terpinolene (6.4), limonene (4.8), β-maaliene (3.7), shyobunone (3.2), α-pinene (3.1)	Saudi Arabia	[9]
*A. nanschanica*	AP	Terpenoids (70.86), thujone (21.3), heptadiene (16.52), linalool (10.94), 1, 8-cineole (9.43), camphor (6.66)	Tibetan plateau	[10]
*A. annua*	AP	Artemisia ketone (30.7), camphor (15.8)	Bosnia	[11]
*A. vulgaris*	L & B	L: Germacrene D (25), caryophyllene (20), alpha-zingiberene (15), borneol (11) B: 1,8-Cineole (32), camphor (16), borneol (9), caryophyllene (5)	Erie, Pennsylvania	[12]
*A. ciniformis*, *A. oliveriana*, *A. turanica*	AP	*A. ciniformis:* Camphor (36.9), 1,8-cineole (20.3), *trans*-pinocarveol (14.7) *A. oliveriana:* Borneol (9.5), eugenol (8.8), spathulenol (7.7) *A. turanica*: 1,8-Cineole (37.7), α-thujone (26.7), *cis*-chrysanthenol (15.3)	Iran	[13]
*A. absinthium*	AP	Myrcene (8.6–22.7), *cis*-chrysanthenyl acetate (7.7–17.9), dihydrochamazulene isomer (5.5–11.6), germacrene D (2.4–8.0), β-thujone (0.4–7.3), linalool acetate (trace-7.0), α-phellandrene (1.0–5.3), linalool (5.3–7.0)	Tajikistan	[14]
*A. absinthium*	FH	*Trans*-sabinyl acetate (45.2), (*cis*+ *trans*) thujones (12.3)	Vilnius, Lithuania	[15]
*A. kermanensis*	S, L & F	S: Camphor (20.3), 1,8-cineole (11.2) L: Selin-11-en-4-alpha-ol (18.6) F: Soborneol (17.1), santolina alcohol (10.6)	Iran	[16]
**Chemical Analysis in 2013**				
*A. deserti*	AP	Camphor (20.1), *trans*-thujone (17.8), 1,8-cineole (10.1)	Iran	[17]
*A. nilagirica*	AP	α-Thujone (36.35), β-thujone (9.37), germacrene D (6.32), 4-terpineol (6.31), β-caryophyllene (5.43), camphene (5.47), borneol (4.12)	India	[18]
*A. herba*-*alba*	AP	Camphor (39.1), chrysanthenone (15.0), *cis*-thujone (7.8)	Tunisia	[19]
*A. lehmanniana*	AP	Camphor (45.6), 1,8-cineole (24.8), camphene (6.8), β-thujone (6.6)	Iran	[20]
*A. dracunculus*	AP	Methyl chavicol (84.83), *trans*-ocimene (3.86), z-beta-ocimene (3.42)	Iran	[21]
*A. arborescens*	AP	Chamazulene (31.9), camphor (25.8)	Tunisia	[22]
*A. absinthium*	L	Borneol (18.7 & 16.7), methyl hinokiate (11.9 & 12.9), isobornyl acetate (4.0 & 4.7), beta-gurjunene (3.8 & 4.4), caryophyllene oxide (3.7 & 4.3)	India	[23]
*A. annua*	AP	Artemisia ketone (35.7), alpha-pinene (16.5), 1,8-cineole (5.5)	Serbia	[24]
*A. alba*	AP	Artemisia ketone (23.7), 1,8-cineole (15.2)	Serbia	[25]
*A. caerulescens* subsp. *densiflora*	AP	Terpinen-4-ol (22), α -terpineol (3.02)	Italy	[26]
*A. chamaemelifolia*	AP	Pelor population: 1,8-Cineole (31.82) Kandovan population: Artemisia ketone (12.27), camphor (17.21), borneol (13.50), davanone D (28.44), davanone (28.88) at the 50% flowering stage Gadok population: Chrysanthenone (18.14)	Iran	[27]
*A. anomala*	AP	Camphor (18.3), 1,8-cineole (17.3), β-caryophyllene oxide (12.7), borneol (9.5)	China	[28]
*A. phaeolepis*	AP	Eucalyptol (11.30), camphor (8.21), terpine-4-ol (7.32), germacrene D (6.39), caryophyllene oxide (6.34), caryophyllene (5.37)	Tunisia	[29]
*A. indica*	AP	Artemisia ketone (42.1), germacrene B (8.6), borneol (6.1), *cis*-chrysanthenyl acetate (4.8)	India	[30]
*A. absinthium*	L & F	Leaves: Chamazulene (30.41), β-thujone (25.75), bornan-2-one (17.33) Flowers: Chamazulene (29.9),β-thujone (19.66), camphor (16.16)	Tunisia	[31]
*A. armeniaca*, *A. incana*	AP	*A. armeniaca:* α–Pinene (10.7), nonadecane (10.0), 6,10,14-trimethyl-2-pentadecanone (9.4), spathulenol (7.8), Z-verbenol (5.8). *A. incana:* Camphor (20.4), 1,8-cineol (10.3), Z-verbenol (8.7), β-thujone (8.3), α-thujone (5.6)	Iran	[32]
**Chemical Analysis in 2014**				
*A. nilagirica*	L	Camphor (32.56), borneol (12.59), caryophyllene (9.6), β-pinene (9.4), β-transocimene (6.14), germacrene-D (5.34)	India	[33]
*A. herba*-*alba*	AP	Camphor (17–33), α-thujone (7–28), chrysanthenone (4–19)	Algeria	[34]
*A. austriaca*	AP	Camphor (15.88), 1,8-cineole (10.75), camphene (3.53)	Iran	[35]
*A. frigida*	AP	*Cis*-rho-menth-2-en-1-ol (20.8), 1,8-cineole (12.0), borneol (10.2), lavandulol (9.3), camphor (6.9), bicyclogermacrene (5.5)	China	[36]
*A. argyi*	AP	Eucalyptol (22.03), β-pinene (14.53), β-caryophyllene (9.24),(-)-camphor (5.45)	China	[37]
*A. hedinii*	AP	1,8-cineol (16.53), camphor (15.20), dehydrosesquicineol (13.59)	Qinghai-Tibetan Plateau	[38]
*A. absinthium*	AP	Sabinene (24.49), sabinyl acetate (13.64), α-phellandrene (10.29)	Serbia	[39]
**Chemical Analysis in 2015**				
*A. herba*-*alba*	AP	*Cis*-chrysanthenol (13.83), 1, 8-cineole (12.84), *cis*-limonene (12.57), α-terpinenol (6.97), γ-muurolene (4.50)	South Jordan	[40]
*A. mongolica*	AP	Eucalyptol (39.88), (S)-*cis*-verbenol (14.93), 4-terpineol (7.20), camphor (6.02), α-terpineol (4.20)	China	[41]
*A. schimperi*, *A. abyssinica*, *A. afra*, *A. absinthium*	L	*A. schimperi*, *A. afra*, *A. abyssinica*: Yogomi alcohol (13.5–37.6), artemisyl acetate (12.7–35.5), artemisia ketone (2.3–13.2). *A. absinthium*: Camphor (21.2–28.3), davanone (21.3–26.5) *A. absinthum* (Europe): β-Thujone (42.3–66.4), chamazulene (11.3–24.2)	Ethiopia	[42]
*A. maderaspatana* (syn. *Grangea maderaspatana*)	AP	α-Humulene (46.3), β-caryophyllene (9.3), alpha-copaene (8.2), β-myrcene (4.3), Z(E)-alpha-farnesene (3.7), calarene (3.5)	India	[43]
*A. dracunculus*	AP	1,8-cineole (35.88), camphor (32.28), camphene (9.13), bomeol (7.07), thymene (3.31), terpinen-4-ol (3.26)	Turkey	[44]
*A. stolonifera*	AP	Eucalyptol (32.93), β-pinene (8.18), camphor (6.12), terpinen-4-ol (6.11)	China	[45]
*A. annua*	AP	α-Pinene (7.33), camphene (5.68), sabinene (4.78), β-myrcene (22.41), 1,8-cineole (17.17), camphor (20.41)	Iran	[46]
*A. herba*-*alba*	AP	α-Thujone (trace-47.1), camphor (5.6–30.0), chrysanthenone (trace-13.5), β-thujone (trace-9.2), 1,8-cineole (4.1–11.4)	Southern Algeria	[47]
*A. annua*	AP	Camphor (17.74), α-pinene (9.66), germacrene D (7.55), 1,8-cineole (7.24), β-caryophyllene (7.02), artemisia ketone (6.26)	Romania	[48]
*A. absinthium*	AP	α & β-Pinene (24.47), pseudolimonen (8.95), geranyl bromide (3.70), terpinolen (2.74), α & β-fellandrene (2.38)	Romania	[49]
*A. campestris* subsp *campestris*	F, L, S	Germacrene D (24.2, 28.0, 27.8), β-caryophyllene (6.5, 5.9, 4.2), γ-humulene (4.9, 6.2, 8.2), (Z)-falcarinol (19.0 & 38.8)	Poland	[50]
*A. herba*-*alba*	AP	1,8-cineole (20.1), α-thujone (25.1), β-thujone (22.9), camphor (10.5)	Jordan	[51]
**Chemical Analysis in 2016**				
*A. stricta*	AP	Capillene (41.6), spathulenol (14.6), β-caryophyllene (13.4)	India	[52]
*A. campestris*	AP	Pinene (18.65), β-pinene (16.78), β-myrcene (17.34), germacrene D (10.34)	Algeria	[53]
*A. stelleriana*	AP	1,8-Cineole (29.5), germacrene D (5.6), vulgarone B (3.1), davanone B (3.0), artedouglasia oxides (22.5: A-8.0; B-4.0; C-5.5; D-5.0)	India	[54]
*A. aucheri*	AP	Linalool (20), camphor (18)	Iran	[55]
*A. sieberi*, *A. aucheri*	AP	*A. aucheri*: Myrcene (19.83), linalool (17.98), lavandulol (7.30), bornyl acetate (6.72), E-nerolidol (6.28), davanone (5.46), 1,8-cineole (4.51), borneol (4.38) *A. sieberi*: Camphor (29.50), *cis*-thujone(22.58), 1,8-cineole (12.91), *trans*-thujone (10.60), camphene (5.05)	Iran	[56]
*A. proceriformis*	AP	Thujone (66.9)	Argentina	[57]
*A. argyi*	AP	Eucalyptol (16.2), β-pinene (14.3), camphor (14.0), artemisia ketone (13.9), α-pinene (11.1)	Tianjin-R China	[58]
*A. turanica*	L	1,8-Cineole (34.2),α-thujone (25.5)	Iran	[59]
*A. ilagirica* var. *septentrionalis*	AP	Artemisia ketone (62.6), artemisia alcohol (3.7), perillene (3.1), β-caryophyllene (3.5), α-muurolol (3.5), δ-cadinene (2.1)	India	[60]
*A. vulgaris*	AP	Artemisia ketone (6.77–29.38), *trans*-caryophyllene (6.22–6.94),1,8-cineole (4.75–5.13), p-cymene (7.60), yomogi alcohol (5.48)	India	[61]
*A. herba alba*	AP	β-Thujone (41.9), α-thujone (18.4), camphor (13.2)	Tunisia	[62]
*A. vulgaris*	AP	Davanones (13.8–45.5, six oils), germacrene D (9.1–30.5, four oils), 1,8-cineole (16.4, one oil), camphor (18.9, one oil), *trans*-thujone (8.9 and 10.9, two oils), *cis*-chrysanthenyl acetate (10.4, one oil)	Lithuania	[63]
*A. judaica*	AP	Piperitone (30.4), camphor (16.1), ethyl cinnamate (11.0)	Jordan	[64]
*A. scoparia*	AP	p-Cymene (0.6–15.2), limonene (0.1–6.3), α-pinene (0.2–10.1), β-pinene (0.4–8.9), *trans*-beta-ocimene (0.3–5.4), caryophyllene (4.6–13.8), germacrene D (11.5–40.3), spathulenol (4.0–11.7), caryophyllene oxide (4.3–15.6)	Buryatia and Mongolia	[65]
*A. sieberi*	AP	Hydrodistillation: Camphor (22.0), 1,8-cineole (19.3), *cis*-davanone (15.0), camphene (4.6), terpinene-4-ol (3.2) Microwave assisted hydrodistillation: *Cis*-davanone (29.8), camphor (20.8), 1,8-cineole (13.8), geranyl acetate (5.7), terpinene-4-ol (3.0)	Iran	[66]
**Chemical Analysis in 2017**				
*A. anethoides*	AP	1,8-cineole (36.54), terpinen-4-ol (8.58), 2-isopropyltoluene (6.20), pinocarveol (5.08)	China	[67]
*A. monosperma*, *A. scoparia*, *A. judaica*, *A. sieberi*	AP	*A. sieberi* and *A. judaica*: Spathulenol 30.42 and 28.41, resp.) *A. monosperma*: Butanoic acid (17.87) *A. scoparia*: Acenaphthene (83.23)	Saudi Arabia	[68]
*A. nilagirica*	AP	β-Thujone (33.78), germacrene-D (9.31), β-thujone (6.01), caryophyllene (5.86), caryophyllene oxide (6.17), borneol (2.16)	India	[69]
*A. absinthium*	L	Camphor (19.0), E-caryophyllene (9.3), eucalyptol (6.8), germacrene D (6.7), α-cadinol(6.5)	Brazil	[70]
*A. campestris*	A	β-pinene (12.0), spathulenol (10.8), α-pinene (7.5), limonene (7.0), o-cymene (5.4)	Morocco (Tigri-Tendrara)	[71]
*A. herba*-*alba*	L	α-Thujone (37.9), germacrene D (16.5), 1,8-cineole (8.4), β-Thujone (7.8)	Tunisia	[72]
*A. dracunculus*	AP	p-Allylanisole (84.00), ocimene (e)-β (7.46), ocimene (z)-beta (6.24), limonene (1.42)	Iran	[73]
